# Abuse-related trauma forward medical care in a randomly sampled nationwide population

**DOI:** 10.1097/MD.0000000000005214

**Published:** 2016-10-28

**Authors:** Cheng-Maw Ho, Chih-Hsin Lee, Jann-Yuan Wang, Po-Huang Lee, Hong-Shiee Lai, Rey-Heng Hu, Jin-Shing Chen

**Affiliations:** a Department of Surgery; b College of Medicine, National Taiwan University; c Division of Pulmonary Medicine, Wanfang Hospital, Taipei Medical University; d Department of Internal Medicine; e Department of Traumatology, National Taiwan University Hospital; f School of Medicine, Taipei Medical University, Taipei, Taiwan.

**Keywords:** abuse, trauma, NHIRD

## Abstract

Abuse-related trauma remains a global health issue. However, there is paucity in nationwide reports. We aim to estimate the incidence of abuse-related trauma forward medical care and identify its characteristics and clinical course in Taiwan. Patients with trauma between 2005 and 2007 that occurred 3 months before or after a diagnosis of abuse were identified from a randomly sampled nationwide longitudinal health insurance database of 1 million beneficiaries. The patients’ demographic data, injury pattern, and medical resource utilization were measured, stratified by age and sex, and compared using chi-square test. Risk factors of next trauma event were identified using Cox regression analysis. Ninety-three patients (65 females) were identified (mean age, 20.6 ± 16.3 years), including 61.3% under 18 years of age. For the first trauma event, 68 patients (73.1%) visited the emergency room, 63 (67.7%) received intervention, and 14 (15.1%) needed hospital care. Seven (7.5%), all less than 11 years old, had intracranial hemorrhage and required intensive care. Thirty-three (35.5%) left with complications or sequelae, or required rehabilitation, but all survived. Of the 34 victims of sexual abuse, 32 were aged less than 18 years. Men received more mood stabilizers or antipsychotics (50.0% vs 10.7%, *P* = 0.030) and reeducative psychotherapy (25.0% vs 0, *P* = 0.044). Risk factors for a next trauma event were injury involving the extremities (hazard ratio [HR]: 5.27 [2.45–11.33]) and use of antibiotics (HR: 4.21 [1.45–12.24]) on the first trauma event. Abuse-related trauma has heterogeneous presentations among subgroups. Clinicians should be alert in providing timely diagnosis and individualized intervention.

## Abstract 2

Supplemental Digital Content is available in the text

## 1 Introduction

Violent abuse is a global issue and the estimated prevalence is that 1 in 4 children has been physically abused; one in 5 girls has been sexually abused; and 1 in 3 women has been a victim of physical and/or sexual intimate partner violence at some point in her lifetime.^[1]^ The prevalence varies between regions and even within countries.^[2]^ The estimated rate of violent abuse is not decreased over the years or even increased in some populations,^[^3–5^]^ resulting in great social and economic burden.^[6]^ For example, in the United States, data indicate that interpersonal violence is among the leading diseases and injuries contributing to premature mortality,^[^7 8^]^ and estimates of the costs reach 3.3% of the gross domestic product.^[9]^

Although the majority of violence is nonfatal, the resultant injuries including mental and reproductive health problems (such as sexually transmitted diseases [STDs]) can last for years and may cause permanent disability.^[10]^ Trauma injury associated with abusive violence, therefore, is an important public health issue. Studies on abuse-related trauma can be helpful in understanding the pattern of this kind of injury in a society, guide policy decision-making, and ensure timely management and protection. The signs of abuse-related trauma were often overlapped with those caused by other trauma etiologies.^[^11 12^]^ There is actually very little about this in literature^[13]^ despite the fact that many of these victims will present to the local trauma service, and clinicians at the frontlines will serve as the primary caregiver during their initial management. Clinicians need to be aware of the issues around domestic violence so they can learn how to appropriately direct treatment for these victims.

As the mandatory universal health insurance program offers comprehensive medical care coverage, the National Health Insurance (NHI) of Taiwan has covered up to 99% of residents (including Taiwanese indigenous people) for several years since 1996.^[14]^ The Taiwanese healthcare system is characterized by good accessibility, comprehensive population coverage, short waiting times, and low patients’ own expense.^[15]^ With a longitudinal follow-up of more than 20 million subjects and validated diagnoses of catastrophic illness,^[^16 17^]^ the National Health Insurance Research Database (NHIRD) provides very suitable research material to explore outcomes of rare diseases or clinical entities.

The present study aimed to analyze the incidence, characteristics, and outcomes of patients of abuse-related trauma seeking medical care in Taiwan using the randomly sampled longitudinal cohort information of the NHIRD.

## 2 Methods

The institutional review board of National Taiwan University Hospital, Taipei, Taiwan approved this study (NTUH REC: 201212001W). As a retrospective study using an encrypted database, the institutional review board waived the need for informed consent.

### 2.1 Data source

The Longitudinal Health Insurance Database (LHID), a subset database of the NHI program, contained the entire original claims data from 1996 to 2010 of 1,000,000 beneficiaries randomly sampled from the year 2005 Registry for Beneficiaries of the NHI program. To balance the need between the enough selected patient number for analysis and ensuring adequate follow-up duration (at least 3 years) after abuse-related trauma, we make a cut-off point at December 31, 2007 so the study encompasses 3-year period (January 2005 to December 2007) with at least 3-year follow-up duration.

### 2.2 Patient selection

Patients who had any record with the diagnosis of trauma (International Classification of Diseases, Ninth Revision, Clinical Modification [ICD-9-CM] 800–999) between January 1, 2005 and December 31, 2007 were identified. Among them, those whose trauma was considered abuse-related were further selected based on the presence of any documentation of the compatible diagnoses of abuse in the adjacent 3 months. The association between abuse and trauma were time sequential and might not be directly causal as the details of medical records were not accessible in the claim dataset. For each patient, the demographic data, laboratory tests, medications, and clinical procedures/interventions during the first episode of abuse-related trauma, as well as the outcomes, were retrieved from the LHID, whatever the medical management for this very patient in different hospitals or clinics was reimbursed.

The compatible diagnoses of abuse included ICD-9-CM code for observation and evaluation of suspected abuse and neglect (V71.81), child maltreatment syndrome (995.5), child abuse (995.50, 995.59), child psychological abuse (995.51), child neglect (995.52), child sexual abuse (995.53), child physical abuse (995.54), shaken infant syndrome (995.55), adult maltreatment or abuse (995.80, 995.85), adult physical abuse (995.81), adult psychological abuse (995.82), adult sexual abuse (995.83), and adult neglect (995.84).

### 2.3 Demographic data

Demographic information including sex, age, underlying comorbidity (ie, diabetes mellitus, chronic obstructive pulmonary lung disease, end-stage renal disease, autoimmune disorder, acquired immunodeficiency syndrome, and malignancy), and low income were collected as in a previous report.^[18]^ Severe trauma was noted if the patient had a catastrophic illness certified by the Bureau of National Health Insurance for an Injury Severity Score of 16 or more.

### 2.4 Imaging and laboratory studies, medications, procedures, and interventions

All reimbursed items were reviewed and recorded: imaging studies, including radiographic, sonographic, and magnetic resonance imaging; laboratory tests, including STD (Venereal Disease Research Laboratory, antibodies against chlamydia, human immunodeficiency virus, and herpes simplex virus), viral hepatitis (hepatitis B virus and hepatitis C virus), and urine pregnancy test; medications, including analgesics, antibiotics, and psychiatric drugs; procedures, including wound treatment, debridement, incision and drainage, blood transfusion, abortion, baby delivery, surgeries, lumbar puncture, slit lamp examination, eye ground examination, clavicle fixation, or extremity splint; and psychiatric interventions, including supportive, reeducative, behavior, intensive, activity, family, or group therapy.

The trauma region was identified by the diagnostic code or relevant imaging studies. The trauma character was further defined as blunt for contusion or concussion; tissue defect for open wound, hemorrhage, burn, fracture, and visceral perforation; or unspecified if not documented.

### 2.5 Follow-up and outcome

The patients were followed-up until December 31, 2010, withdrawal of the health insurance, or death. Death was considered if a subject canceled health insurance and had no medical records in the subsequent 1 year. Rehabilitation, complications, and sequelae were recorded by interpreting the reimbursed codes of diagnosis, medications, interventions, and procedures. New trauma event was defined as the occurrence of trauma that was different from the abuse-related trauma in ICD-9-CM code beyond 3 months after the first event of abuse-related trauma. It was retrieved from the LHID and was confirmed by the compatible interventions that were reimbursed for diagnostic or therapeutic reasons.

### 2.6 Statistical analysis

Data were expressed as means ± standard deviation, median, range, or number (percentage), as appropriate. The Student *t* test or chi-square test was used for intergroup comparison. The time-to-event curves for new trauma event stratified by different risk factors were generated using the Kaplan–Meier method and compared using the log-rank test. The Cox's proportional hazard model was used to identify independent prognostic factors of the next trauma event. Statistical significance was set at a 2-sided *P* < 0.05. All analyses were performed using the Statistical Package for Social Sciences (SPSS) version 18.0 (IBM Corporation, Armonk, NY).

## 3 Results

### 3.1 Patient selection, incidence, and general demographics

**Figure 1:**
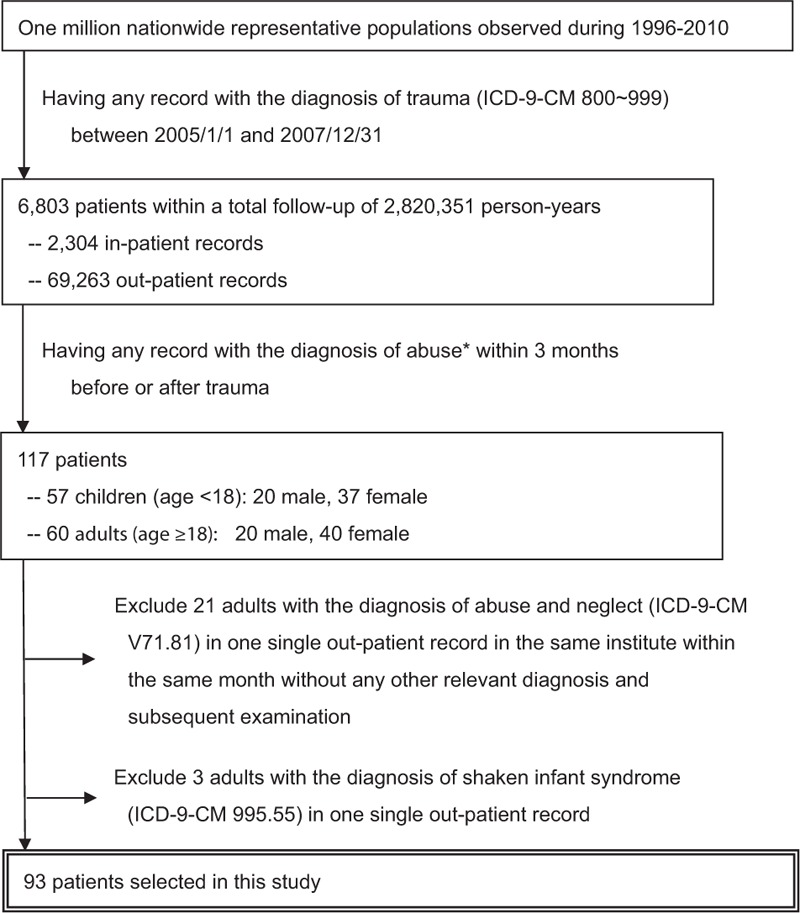
Schematic representation of the patient selection process.∗The diagnoses of abuse included ICD-9-CM code for observation of abuse and neglect (V71.81), child maltreatment syndrome (995.5), child abuse (995.50, 995.59), child psychological abuse (995.51), child neglect (995.52), child sexual abuse (995.53), child physical abuse (995.54), shaken infant syndrome (995.55), adult maltreatment or abuse (995.80, 995.85), adult physical abuse (995.81), adult psychological abuse (995.82), adult sexual abuse (995.83), and adult neglect (995.84). ICD-9-CM = International Classification of Diseases, ninth revision, clinical modification.

From a total follow-up of 2,820,351 person-years between 2005 and 2007, 6803 potentially eligible trauma patients were identified (Fig. 1), including 117 with abuse-related trauma. Twenty-one (all aged at 60s or 70s) were excluded due to the unusual diagnostic code combination of ICD-9-CM V71.81 (observation and evaluation of suspected abuse and neglect) and 780.81 (chronic fatigue syndrome) in a single medical institute within 1 month and no further relevant intervention and follow-up visit could be traced. Another 3 adults were excluded due to irrelevant diagnostic code (ICD-9-CM 995.55; shaken infant syndrome) without subsequent examination or follow-up. The remaining 93 patients were enrolled in this study.

Patient characteristics, clinical course, and outcome were shown in Tables 1 and 2. The 93 patients (mean age, 20.6 ± 16.3 years; 65 females) consisted of 36 adults (28 females) and 57 children (age <18 years; 37 females), with mean ages of 37.6 ± 12.7 and 9.9 ± 5.6 years, respectively. More than one-third (n = 34, 36.6%) suffered from sexual abuse and most (n = 32) were children (<18 years of age). The estimated incidence based on this random sampled 1 million population claim dataset was 33.0 per million person-years for all abuse, 86.0 for child abuse, and 14.5 for sexual abuse.

**Table 1:**
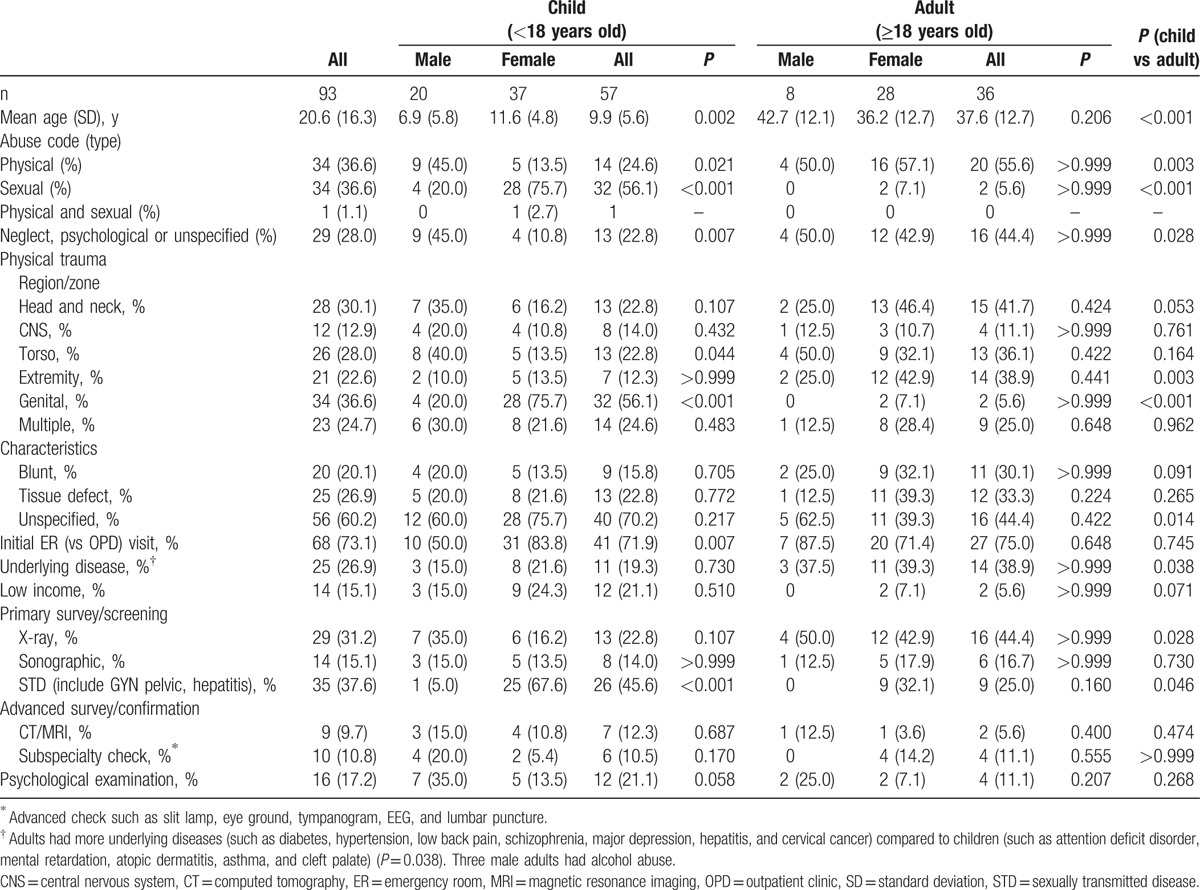
Characteristics of patients with abuse-related trauma.

**Table 2:**
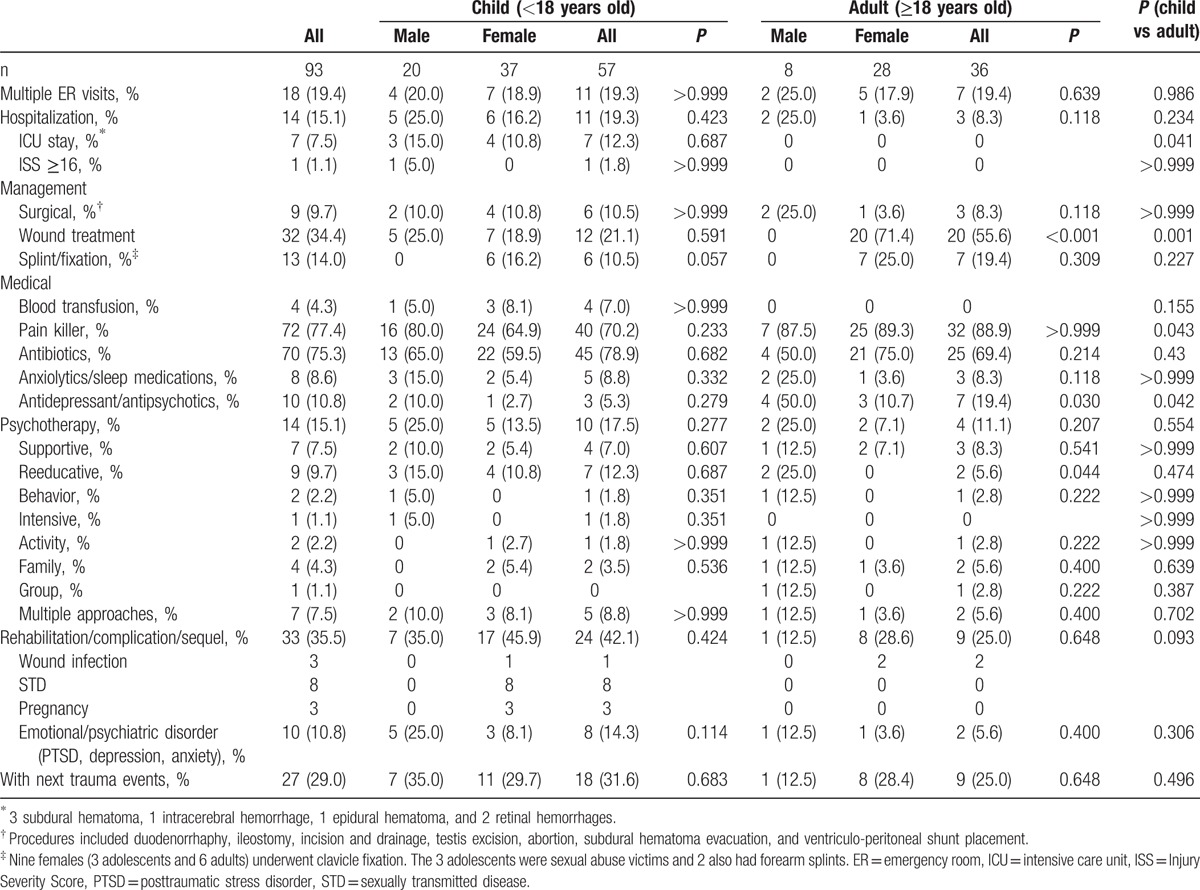
Clinical course and outcomes of abuse-related trauma.

**Figure 2:**
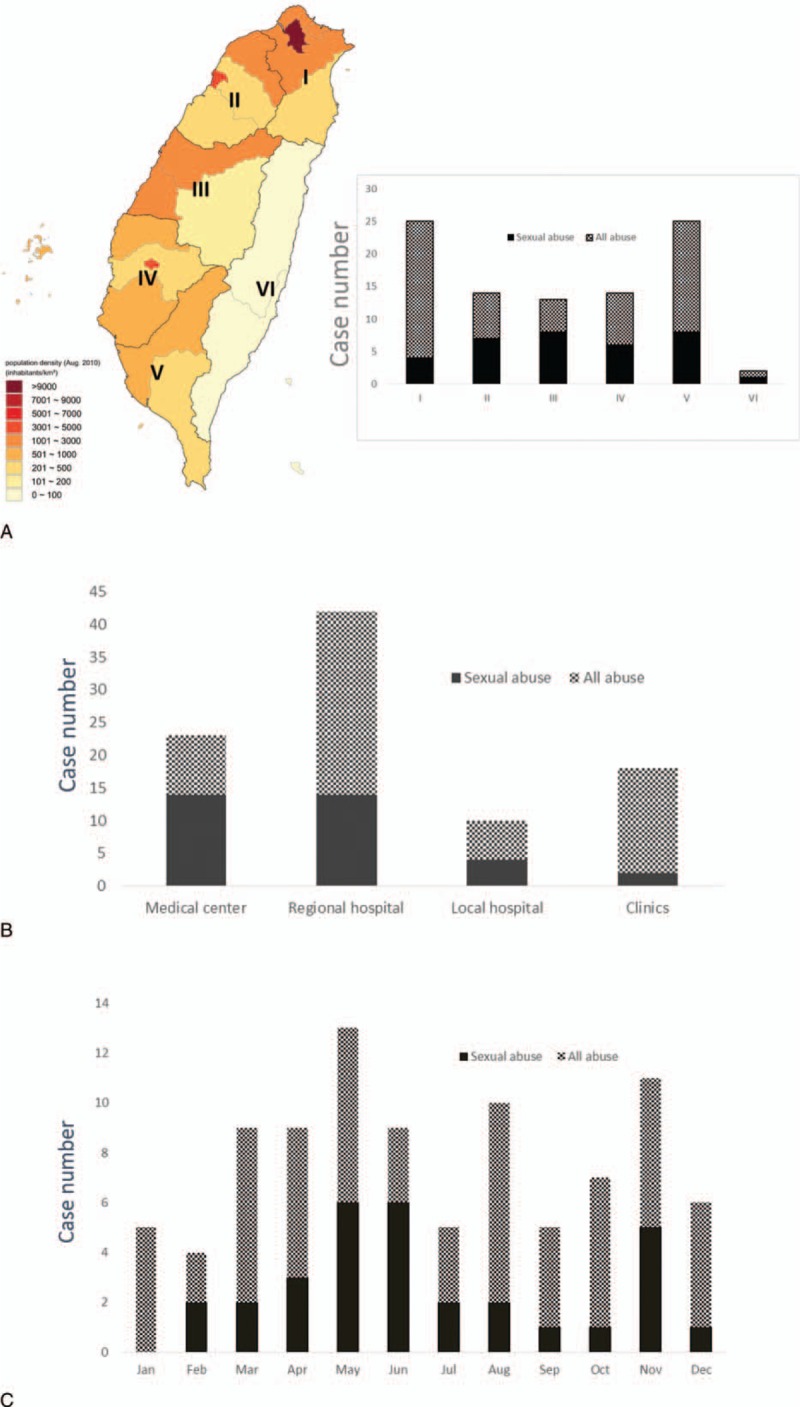
(A) Geographic, (B) first medical resource seeking, and (C) seasonal distribution of abuse-related trauma in Taiwan. Note that at Changhua County located in the middle of Taiwan (zone IV, A), 9 patients were victimized and 8 were managed in one medical center. Seven of them suffered from sexual abuse. Taiwan map (A) was modified from the webpage of Wikimedia Commons and under the Creative Commons Attribution-Share Alike 3.0 Unported license.^[19]^ Roman numerals (I–VI) denote 6 regional divisions (Taipei, Northern, Central, Southern, Kaoping, and Eastern Divisions, respectively) of NHI Administration across Taiwan. Levels of hospitals are defined according to NHI payment system and accredited by Taiwan Joint Commission on Hospital Accreditation. NHI = National Health Insurance.

By geographic distribution, more cases were reported in highly populated west-coasted urban cities than in east-coast areas (Fig. 2A). Most cases were managed in regional hospitals (Fig. 2B). Victimization (from either sexual or all other abuses) occurred more frequently in spring (Mar-May, n = 40) (Fig. 2C).

No patient died of trauma in this cohort during follow-up. Of the 93 patients, 60.2% had unspecified documentation of injury and more so in children than in adults (*P* = 0.014). Trauma was first noted in the emergency room (ER) in 68 (73.1%). Multiple ER visits were noted in 16 (17.2%) cases due to trauma at or after the index trauma date, and 2 (2.2%) due to acute complication of schizophrenia (n = 1) and hydrocephalus-related seizure (n = 1) before the index date. Among the 93 patients with abuse-related trauma, 63 (67.7%) received intervention and 14 (15.1%; 11 children and 3 adults) needed hospital care (Table 1). The indications for admission were neurological or/and intracranial hemorrhage (7 children), burns (2 children with 1 chemical burn), intestinal perforation (1 child who also had neurological injury), fracture (1 adult), and psychological trauma (1 child with sex abuse and 2 adults). The median length of hospital stay was 12.5 days (range, 1–100) and was not different between the adults (9.5 days) and the children (13.0 days) (*P* = 0.592) (Table 2). Thirty-three of the 93 patients had complications/sequelae and needed rehabilitation after the abuse-related trauma. Eight girls were infected by STD and 3 became pregnant after the sexual abuse.

More than one third (36.6%) suffered from physical abuse. Adult patients suffered more physical abuse compared to children (*P* = 0.003) and in the child group, more on boys than on girls (*P* = 0.021). The anatomical regions of physical trauma most often affected were the head and neck, followed by the torso and extremities. Boys were younger in age (*P* = 0.002) and had more torso injury than girls (*P* = 0.044). About one-fourth had multiple anatomical sites of injury. By initial trauma surveys, the adult group had more radiographic examinations than the child group (*P* = 0.028). Screening for STDs was more often performed on the child group (*P* = 0.046) and correlated with more sexual abuse observed (*P* < 0.001).

### 3.2 Complex abuse presentations, trauma mechanisms, and combinations

Among 34 sexual abuse victims, 7 suffered from additional physical injuries [head and neck (n = 2), torso (n = 3), and extremity (n = 4)]. Combination of abuses was more frequently observed in children group. For example, a 4-year-old girl who suffered from abuse-related subdural hematoma was malnourished and had growth delays; a 10-year-old boy victim of multiple abusive physical trauma and chemical burn had previous burn injuries; another 11-year-old boy victim of sexual and physical abuse had toxic encephalopathy and scrotal trauma who subsequently received testis excision and survived. Children were also more likely to be suffered from central nervous system injuries and were observed in the intensive care unit for 1 to 2 days. One of them, a 3.5-year-old boy with an Injury Severity Score of 16 or more, had ICH, duodenal perforation, and transverse colon contusion. He had septic shock and underwent duodenorrhaphy, ileostomy, and drainage of intra-abdominal abscess. He stayed in hospital for 51 days and was discharged for home.

### 3.3 Psychiatric intervention

Most cases were managed conservatively with analgesics, antibiotics, and wound treatment [ice packing (n = 9) and wound cleaning/dressing change (n = 27)] and adults (all women) had more wound treatment than children (*P* = 0.001). Adults (especially men, *P* = 0.030) were more likely to receive mood stabilizing drugs or antipsychotics than children (*P* = 0.042). Among the adults, men received more mood stabilizing medications or anti-psychotics (50.0% vs 10.7%, *P* = 0.030) and re-educative psychotherapy (25.0% vs 0, *P* = 0.044) than women. Fourteen (15.1%) victims underwent psychiatric consultation and psychotherapy, which included reeducative (n = 9, including 7 children), supportive (n = 7), family (n = 4), activity (n = 2), behavior (n = 2), and intensive approach (n = 1). In the 34 sexually abused patients, 4 had psychological consultation/examination and 2 underwent psychotherapy. Ten patients had psychological problems, including 2 for psychosis, anxiety, and 1 for adjustment reaction, posttraumatic stress disorder, aggressive emotion, depression, parent–child problem, and development/language delays.

### 3.4 Next event of trauma and risk factor analysis

Twenty-seven (29.0%) of the 93 patients enrolled in the study suffered from new trauma event beyond three months after the initial abuse-related trauma (Table 2). Among them, nine were adults (male/female = 1/8 and median age of 27.5 years) and 18 were children (male/female = 7/11 and median age of 13.5 years). In term of the second trauma event, only one 3.5-year-old victim of physical abuse was recorded as suspected abuse and neglect (V71.81). Among others (n = 26), no additional coding of abuse was noted along with the further trauma.

The locations of these new trauma events were the extremities in 20, torso in 4, and head and neck in 4. Compared to those without new trauma event, the initial abuse-related trauma were more likely to be located at the torso (44.4% vs 15.2%, *P* = 0.023), extremities (48.1% vs 12.1%, *P* < 0.001) or multiple anatomic sites (44.4% vs 16.7%, *P* = 0.005), and more likely to be blunt injury (21.3% vs 15.2%, *P* = 0.020) (Table 3). These 27 patients were also more likely to receive wound treatment (55.6% vs 25.8%, *P* = 0.006) and antibiotics (85.2% vs 56.1%, *P* = 0.008).

**Table 3:**
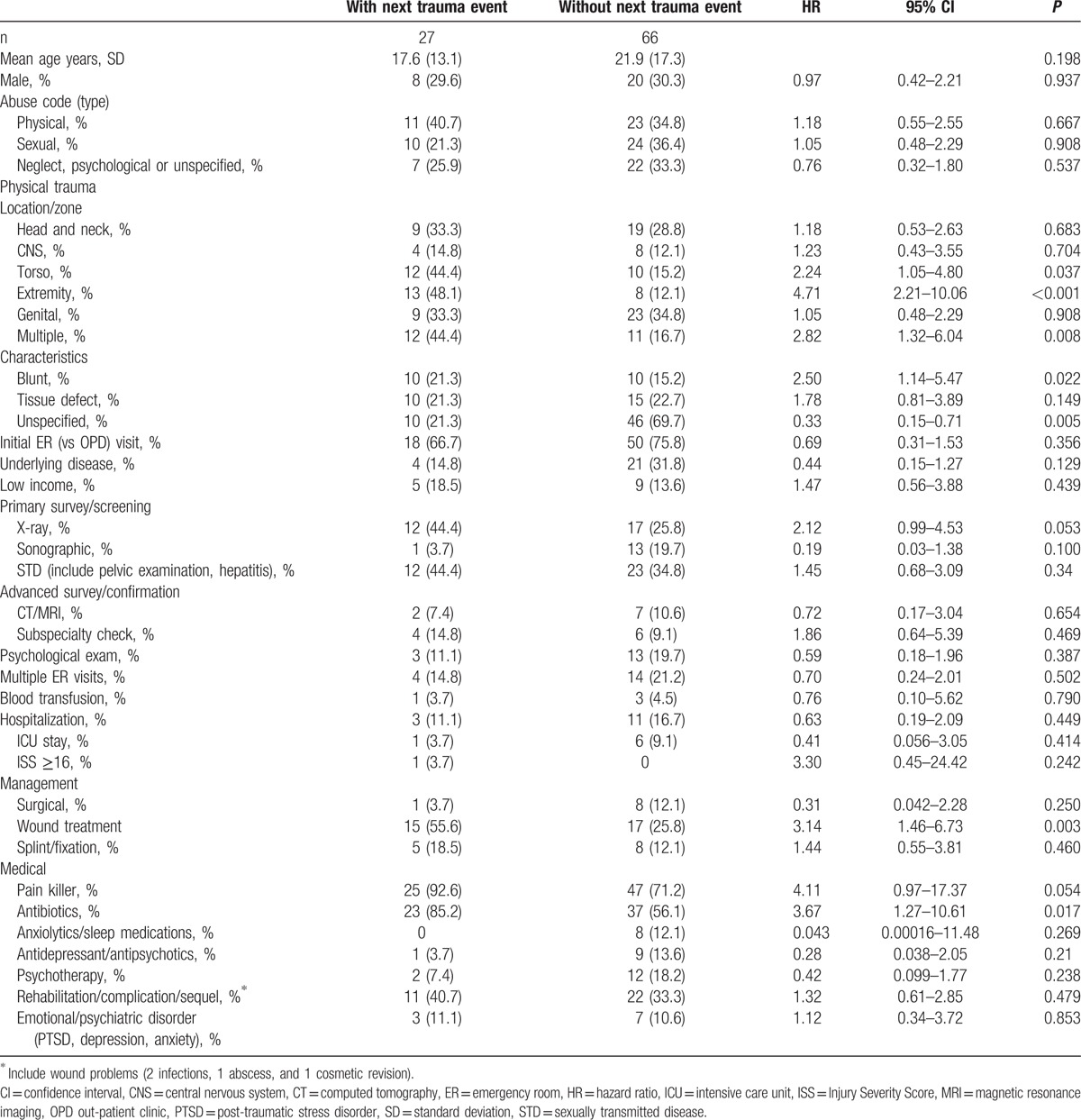
Patients with next trauma events compared to those without.

Cox regression multivariate analysis revealed that the independent risk factors predicting new trauma event were injury at the extremity (hazard ratio [HR]: 5.27 [95% confidence interval] [2.45–11.33]) and use of antibiotics (HR: 4.21 [1.45–12.24]) on the first trauma event (Table 4). The Kaplan–Meier curves for new trauma event were further stratified by these two factors (Fig. S1). In subgroup multivariate analysis, the independent risk factors were injury at the extremity (HR: 8.62 [3.03–24.55]), use of antibiotics (HR: 4.17 [1.18–14.73]), and behavior therapy (HR: 35.97 (3.05–423.90) for children, and injury at the extremity (HR: 5.78 [1.12–29.85]) for adults (Table 4).

**Table 4:**
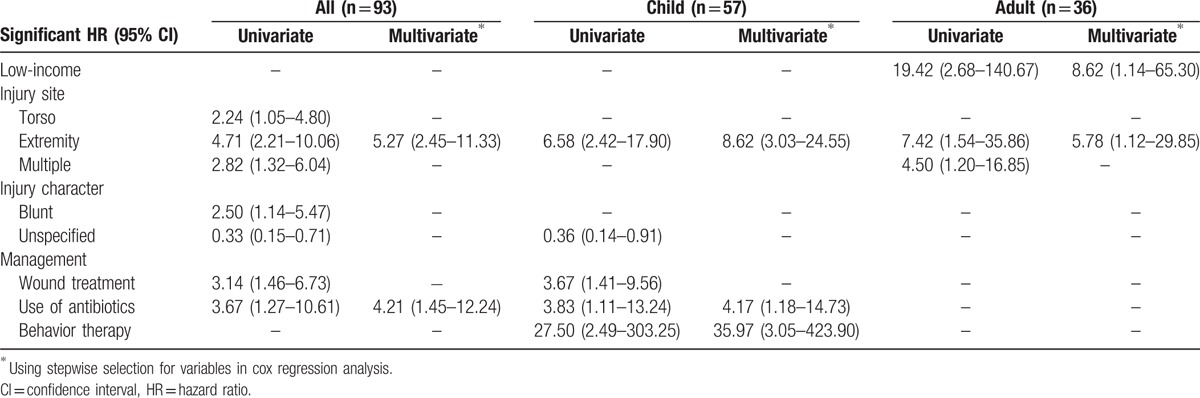
Hazard ratios of risk factors predicting the next trauma event in the 93 patients.

## 4 Discussion

By analyzing the nationwide abuse-related trauma cohort, the present study has 4 main findings. First, the incidence of abuse-related trauma was 33.0 per million person-year in Taiwan. Most of the injuries were due to physical and sexual abuse occurring in urban cities. Most victims of physical abuse were adult women and children, while most victims of sexual abuse were at age below 18 years. Second, over 70% of victims visited ER for medical care and 15% require hospitalization, among the latter half needed intensive care. Two-thirds received intervention (including wound treatment, surgery, or psychotherapy) and over 70% receive analgesics or antibiotics. Third, the injury sites were age dependent. In adults, physical abuse frequently involved the extremities, whereas in children, the head and neck and the torso were affected. Lastly, the risk factors for next trauma event were injury on the extremity (HR: 5.27 [2.45–11.33]) and use of antibiotics (HR: 4.21 [1.45–12.24]) on the first trauma event. These risk factors might be plausible in adolescents or adults (but not in small children) because the underlying violence could be severe enough to cause open wounds (common indications for the use of antibiotics in trauma patients) and arouse the injured to defense against the abuser (injury of extremities) but not to the degree of death, and the injured might carry the residual violent threat (if not clear off) with the increased risk of the next trauma event. The notion that injury of extremities as a risk factor may actually reflect a signal of future vulnerability to a more variety of different trauma events.

A previous study conducted by Chien et al^[20]^ gives a detailed description on hospitalized pediatric population of abuse in Taiwan over a longer time period 1997 to 2009. The present study complements current epidemiologic information on abuse-related trauma injuries in Asia. Though being descriptive in nature and heterogeneous in patient characteristics, this study gives a general picture as well as socioeconomic and medical burden on abuse-related trauma in Taiwan and our result might be generalizable to Asian population (especially influenced by Chinese culture) in the world. A study conducted in Hong Kong reported that the incidence of domestic violence was 1200 to 1400 per 1.7 to 2.4 million, higher than that in present study.^[21]^ However, the participants were the first attendants in the emergency departments of three regional hospitals, rather than general population. In another study conducted in United States utilizing National Trauma Data Bank, Joseph et al^[22]^ reported the prevalence of domestic violence among trauma patients was 5.7 cases per 1000 discharges from trauma center over a 6-year period (2007–2012) by utilizing National Trauma Data Bank. Although the population base was different and the follow-up time of each patient at risk was unrecognized, our result – 0.93 cases (abuse-related trauma) per 1000 ordinary people with a follow-up for at least 3 years (2008–2010) seemed a reasonable estimation.

Indeed, considerable variation among studies regarding vital conceptual and methodological characteristics, which makes the epidemiologic findings vary widely and the attempt of comparison between them difficult.^[23]^ Because the secretive and stigmatizing nature of some types of traumatic events, even data retrieved directly from medical records or from retrospective interviewing may not truly close to the truth. However, we, to our best, utilized the information of laboratory exams, clinical tests, imaging, and psychiatric counseling from the claim database (containing medical orders and management recorded from clinical practice) to objectively illustrate the crude (at least) picture of abuse-related trauma forward medical care in Taiwan (not exclusively based on ICD diagnostic code). The findings of this study provide critical information for public awareness, policy making, and international communication.

Most sexual abuse involves teenage girls. However, less than 12% and less than 6% receive psychological consultation/examination and psychotherapy, respectively. This is even less so nowadays, as patriarchal fetish for female chastity in the Chinese cultural construction of sexual victimization still exists and sexual stigmatization is observed as the most prevailing postabuse trauma among Chinese sexual abuse survivors.^[24]^ Trauma-focused cognitive-behavioral therapy shows good and durable improvement in reducing posttraumatic stress symptoms like anxiety, depression, sexual problems, and dissociation.^[^25 26^]^ It seems very few people received either medical or psychotherapy for the emotional impact of suffering abuse in society under Chinese cultural influence. More emphasis should be placed on this subject when dealing with population health and welfare.

This study, although nationwide in scale, consists of enough unique details from the NHIRD to cover case diversity and distribution. Compared to other studies, the results here are consistent with the findings that abusive head trauma is the most common cause of morbidity in physically abused infants.^[^26 27^]^ Rare presentations in literature, such as duodenal perforation,^[^28 29^]^ burn,^[30]^ or scrotal trauma^[31]^ in child abuse are also observed in this cohort. Understanding the diverse presentations of abuse-related trauma can alert physicians when they encounter unexplained unusual symptoms or signs in the first setting. Early intervention at this time point may reduce the next episode of trauma. Prompt intervention by a social worker or mental specialist may hasten the healing of psychological trauma and reduce the likelihood of mental illness in the long run.^[^32 33^]^

This study has limitations. The incidence may be underestimated if the trauma patients did not seek medical help and if correct diagnostic coding was not made possibly due to the victimization and social stigma that cause reluctance to disclose the trauma mechanism. Information is also lacking on educational status, race, the relationship between the victims and the abusers, psychological- or neglect-related trauma, and professional tests/questionnaire (such as Traumatic Events Survey) in the Taiwan NHIRD.^[34]^ Furthermore, it is unknown whether there is a difference in willingness to report abuse in urban and rural areas. A firm, nationwide reporting system is necessary to understand the true incidence of abuse-related trauma.^[^22 35^]^

## 5 Conclusions

Abuse-related trauma had a wide variety of presentation patterns among different subgroups. Clinicians should be aware of this and be alert so as to provide timely diagnosis and early individualized intervention.

## Supplementary Material

Supplemental Digital Content
