# Abuse-related trauma forward medical care in a randomly sampled nationwide population: Erratum

**DOI:** 10.1097/MD.0000000000008580

**Published:** 2017-11-03

In the article, “Abuse-related trauma forward medical care in a randomly sampled nationwide population”,^[1]^ which appeared in Volume 95, Issue 43 of *Medicine*, Dr. Chih-Hsin Lee's affiliation should have appeared as Division of Pulmonary Medicine, Department of Internal Medicine, Wanfang Hospital and Division of Pulmonary Medicine, Department of Internal Medicine, School of Medicine, College of Medicine, Taipei Medical University, Taipei, Taiwan.
