# Supplementary material for: Abuse-related trauma forward medical care in a randomly sampled nationwide population
Source: Medicine (Baltimore). 2016 Oct 28;95(43):e5214. doi: 10.1097/MD.0000000000005214 (PMC5089111; doi:10.1097/MD.0000000000005214)
Supplement: Supplemental Digital Content [file medi-95-e5214-s001.doc]

**Figure S1**. Kaplan-Meier curve for next trauma event for abuse-related trauma patients stratified according to (A) the injury site on the extremities and (B) use of antibiotics.

**
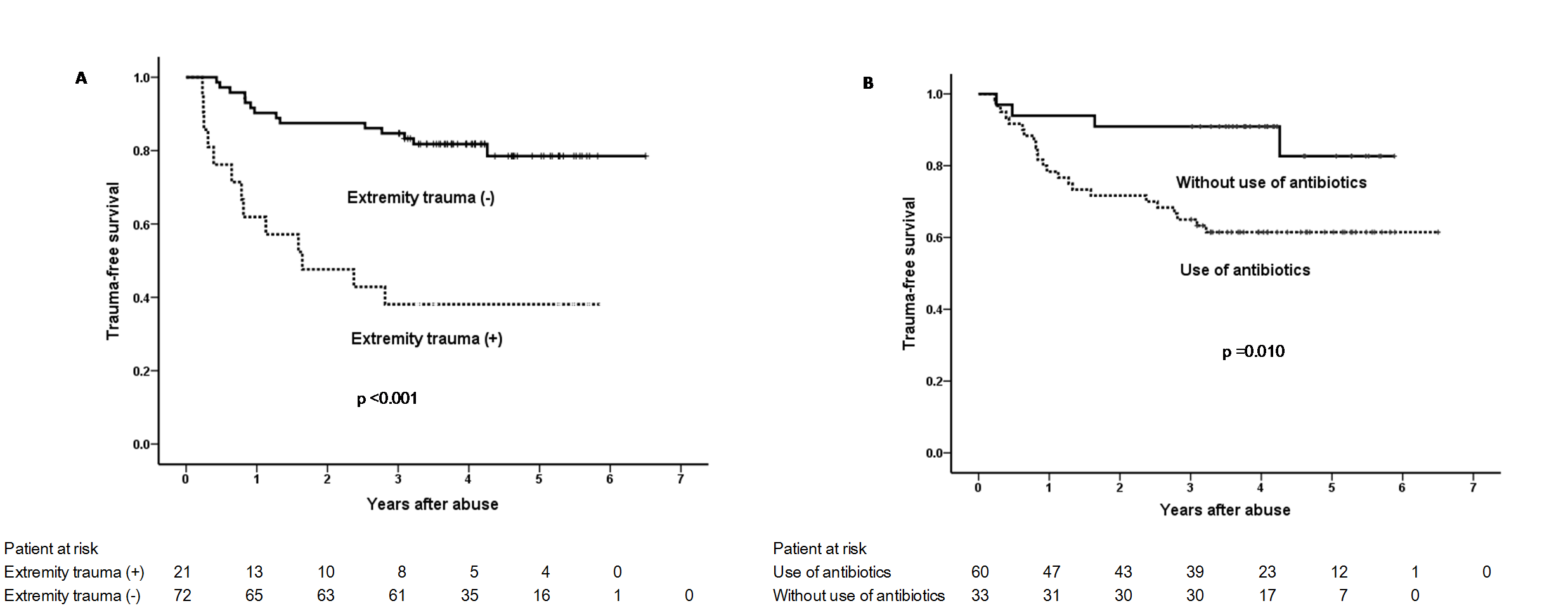

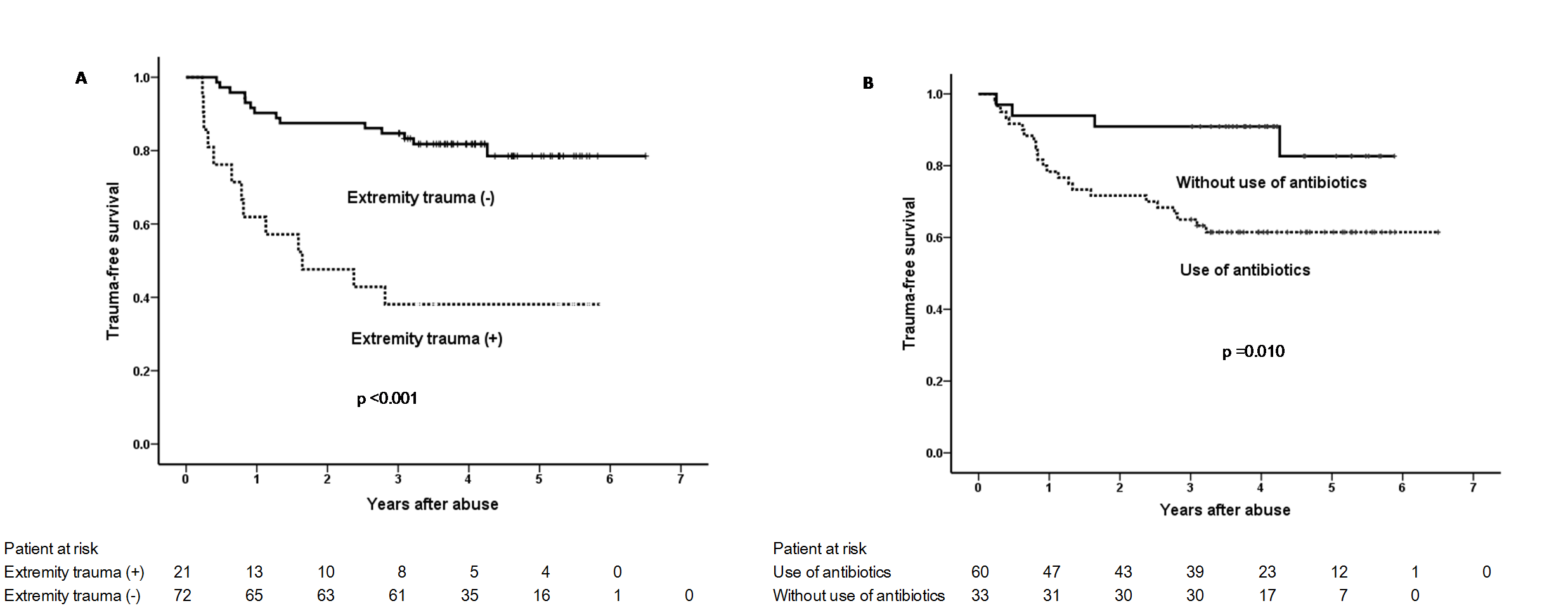
**
